# Kinetics of circulating immunoglobulin M in sepsis: relationship with final outcome

**DOI:** 10.1186/cc13073

**Published:** 2013-10-21

**Authors:** Evangelos J Giamarellos-Bourboulis, Efterpi Apostolidou, Malvina Lada, Ioannis Perdios, Nikolaos K Gatselis, Iraklis Tsangaris, Marianna Georgitsi, Magdalini Bristianou, Theodora Kanni, Kalliopi Sereti, Miltiades A Kyprianou, Anastasia Kotanidou, Apostolos Armaganidis

**Affiliations:** 1 4th Department of Internal Medicine, University of Athens, Medical School, ATTIKON University Hospital, 1 Rimini Street, 12462 Athens, Greece; 2 Intensive Care Unit, Bodosakeio General Hospital, 50200 Ptolemadia, Greece; 3 2nd Department of Internal Medicine, Sismanogleion General Hospital, 1 Sismanogleiou Street, 15127 Athens, Greece; 4 1st Department of Internal Medicine, 'G. Gennimatas’ General Hospital, 154 Mesogeion Street, 11527 Athens, Greece; 5 Department of Medicine and Research Laboratory of Internal Medicine, University of Thessaly, Medical School, 22 Papakiriazi, 41222 Larissa, Thessaly, Greece; 6 2nd Department of Critical Care Medicine, University of Athens, Medical School, ATTIKON University Hospital, 1 Rimini Street, 12462 Athens, Greece; 7 Department of Urology, Lamia General Hospital, Old National Road, 35100 Lamia, Greece; 8 1st Department of Critical Care Medicine, University of Athens, Evangelismos General Hospital, 35 -37 Ispilantou Street, 10672 Athens, Greece

## Abstract

**Introduction:**

The aim of this study was to investigate the kinetics of immunoglobulin M (IgM) during the different stages of sepsis.

**Methods:**

In this prospective multicenter study, blood sampling for IgM measurement was done within the first 24 hours from diagnosis in 332 critically ill patients; in 83 patients this was repeated upon progression to more severe stages. Among these 83 patients, 30 patients with severe sepsis progressed into shock and IgM was monitored daily for seven consecutive days. Peripheral blood mononuclear cells (PBMCs) were isolated from 55 patients and stimulated for IgM production.

**Results:**

Serum IgM was decreased in septic shock compared to patients with systemic inflammatory response syndrome (SIRS) and patients with severe sepsis. Paired comparisons at distinct time points of the sepsis course showed that IgM was decreased only when patients deteriorated from severe sepsis to septic shock. Serial measurements in these patients, beginning from the early start of vasopressors, showed that the distribution of IgM over time was significantly greater for survivors than for non-survivors. Production of IgM by PBMCs was significantly lower at all stages of sepsis compared with healthy controls.

**Conclusions:**

Specific changes of circulating IgM occur when patients with severe sepsis progress into septic shock. The distribution of IgM is lower among non-survivors.

## Introduction

Although initially considered a state of hyperactivity of the innate and adaptive immune systems, it is currently understood that severe sepsis and septic shock are characterized by a functional state of immunoparalysis [1]. This involves not only monocytes and macrophages, but also CD4 lymphocytes and B lymphocytes [2]. Under normal conditions, CD4 lymphocytes orchestrate B lymphocyte responses for the secretion of the polyvalent immunoglobulin M (IgM) antibodies that are of crucial importance for the opsonization and the subsequent rapid clearance of the invading microorganisms [3]. Immunoparalysis of sepsis is characterized by defective B-lymphocyte responses toward low immunoglobulin production [2].

To this end, it was expected that the intravenous administration of immunoglobulin preparations enriched in IgM would be beneficial for patients with severe sepsis and septic shock. On the contrary, most of the conducted randomized clinical trials (RCT) yielded contradictory results [4,5], despite one meta-analysis indicating that IgM preparations significantly decrease the relative risk of death in both adult and child populations [4].

The existing controversies of conducted RCTs may derive from our incomplete understanding of the kinetics of IgM over the time course of sepsis. The current study was designed in order to embed into the changes of circulating IgM levels of patients upon progression to the more severe stages of sepsis in relation with the production of IgM from circulating lymphocytes and with the final outcome.

## Materials and methods

### Study design

This prospective multicenter study was conducted from January 2010 to December 2010 in 27 departments across Greece participating in the Hellenic Sepsis Study Group. The participating departments were 15 intensive care units (ICUs), seven departments of Internal Medicine, two departments of pulmonary medicine, two departments of surgery and one department of urology.

Patients with signs of systemic inflammatory response syndrome (SIRS) either admitted to the emergency department or hospitalized in the general ward or in the ICU were eligible. Written informed consent was provided by the patients or by their first-degree relatives for patients unable to consent. The study protocol was approved by the Ethics Committees of the participating hospitals (Ethics Committee of Alexandra Athens General Hospital; Ethics Committee of 'Aghia Olga’ Athens General Hospital; Ethics Committee of Argos General Hospital; Ethics Committee of ATTIKON University Hospital; Ethics Committee of 'G. Gennimatas’ Athens General Hospital; Ethics Committee of 'G. Gennimatas’ Thessaloniki General Hospital; Ethics Committee of Evangelismos Athens General Hospital; Ethics Committee of Chios General Hospital; Ethics Committee of Ippocrateion General Hospital; Ethics Committee of Laikon Athens General Hospital; Ethics Committee of 'Korgialeneion-Benakeion’ Athens General Hospital; Ethics Committee of Lamia General Hospital; Ethics Committee of Larissa University Hospital; Ethics Committee of Nafplion General Hospital; Ethics Committee of Ptolemaida General Hospital; Ethics Committee of Sismanogleion Athens General Hospital; Ethics Committee of Sotiria Athens General Hospital; Ethics Committee of Sparti General Hospital; Ethics Committee of Thriassion Elefsis General Hospital; and Ethics Committee of Tzaneion Piraeus General Hospital). Each patient was enrolled once.

Inclusion criteria were: (a) age ≥18 years; (b) diagnosis of SIRS, sepsis, severe sepsis or septic shock; and (c) SIRS due to acute pancreatitis or sepsis due to specific infections. These infections were: community-acquired pneumonia (CAP), ventilator-associated pneumonia (VAP), acute pyelonephritis (UTI), acute intra-abdominal infection (IAI) and primary bacteremia (BSI); and (d) first blood sampling within 24 hours from diagnosis.

Exclusion criteria were (a) infection by the human immunodeficiency virus type 1; (b) neutropenia defined as less than 1,000 neutrophils/mm^3^; (c) chronic intake of corticosteroids defined as systemic intake of more than 1 mg/kg of equivalent prednisone for more than one month; and (d) other types of immunodeficiency like organ transplantation, hematologic malignancies and intake of chemotherapy.

SIRS was diagnosed by the presence of at least two of the following [6]: (a) core temperature >38°C or <36°C, (b) P_co2_<32 mmHg or more than 20 breaths/min, (c) pulse rate >90/min, and (d) white blood cells >12,000/mm^3^ or <4,000/mm^3^ or >10% of band forms. Sepsis was defined as any microbiologically or clinically documented infection complicated by SIRS. Patients with sepsis were classified as suffering from uncomplicated sepsis, severe sepsis or septic shock, according to standard definitions [6]. Multiple organ dysfunctions syndrome (MODS) was defined by the same criteria [6]. Acute pancreatitis, CAP, VAP, UTI, IAI and BSI were defined according to standard definitions [7-11].

For each patient a complete diagnostic workup was performed comprising history, thorough physical examination, white blood cell (WBC) count, blood biochemistry, arterial blood gas, blood cultures from peripheral veins and central lines, urine cultures, chest X-ray and chest and abdominal computed tomography if appropriate. If necessary, quantitative cultures of tracheobronchial secretions (TBS) or bronchoalveolar lavage (BAL) were performed and evaluated as previously described [9]. Survival was recorded for 28 days and at hospital discharge. Clinical and demographic data were recorded on a case report form (CRF). All CRFs were monitored by an independent monitor blinded to the study design.

### Blood sampling and laboratory procedure

For all enrolled patients and for 35 healthy volunteers 5 ml of blood was sampled within the first 24 hours from diagnosis. From this volume: (a) 2 ml was collected into sterile, pyrogen- and anticoagulant-free tubes (Vacutainer, Becton Dickinson, Cockeysville, MD, USA) for quantitative measurement of IgM; and (b) 3 ml was collected into EDTA-coated tubes (Vacutainer) for the measurement of the absolute counts of B lymphocytes. From 55 patients and 20 healthy volunteers another 8 ml was collected into heparin-coated tubes (Vacutainer) and used for the isolation of peripheral blood mononuclear cells (PBMCs). For 83 patients, blood sampling was repeated on the day of worsening of sepsis stage. For 30 patients who progressed into septic shock, blood was sampled daily for seven days starting immediately after the start of vasopressors. Tubes were transported by a courier service within the same day to the Laboratory of Immunology of Infectious Diseases of the 4^th^ Department of Internal Medicine at ATTIKON University Hospital of Athens. Tubes were centrifuged and serum was kept frozen at -70°C until assayed. IgM was estimated in duplicate by an enzyme-linked immunosorbent assay (e-Bioscience Inc., San Diego, CA, USA) following the manufacturer’s instructions; the lower detection limit was 20 ng/ml. All estimations were performed and reported by two technicians who were blinded to clinical information.

The central laboratory of the study participates in the UK NEQAS quality control system for leukocyte immunophenotyping (registration number 40926). In this laboratory, the absolute count of B lymphocytes was measured as described elsewhere [12]. Briefly, red blood cells were lysed with ammonium chloride 1.0 mM. White blood cells were washed three times with phosphate-buffered saline (PBS) (pH 7.2) (Merck, Darmstadt, Germany) and subsequently incubated for 15 minutes in the dark with the monoclonal antibody anti-CD19 at the flurochrome fluorescein isothiocyanate (FITC, emission 525 nm, Immunotech, Marseille, France) using fluorospheres (Immunotech) for the determination of absolute counts. One IgG isotypic negative control at the fluorocolor FITC was analyzed for every patient. Cells were analyzed after running through the EPICS XL/MSL flow cytometer (Beckman Coulter, Inc., Miami, FL, USA) with gating for mononuclear cells based on their characteristic forward scatter/side scatter (FS/SS) scattering.

The isolation of PBMCs was limited to 55 patients because these samples should come from patients hospitalized at study sites close to the central laboratory. This allowed the time from blood collection until processing to be less than 30 minutes. As such, PBMCs were studied from patients hospitalized at the ATTIKON University Hospital that is close to the central laboratory of the study. Production of IgM was studied according to a procedure described elsewhere [13]. Heparinized venous blood was layered over Ficoll Hypaque (Biochrom, Berlin, Germany) and centrifuged for 20 minutes at 1400 g. Separated PBMCs were washed three times with ice-cold PBS (pH: 7.2) (Biochrom) and counted in a Neubauer chamber. Their viability was more than 99% as assessed by trypan blue exclusion of dead cells. They were then diluted in RPMI 1640 enriched with 2 mM of L-glutamine, 10% fetal bovine serum (Biochrom), 100 U/ml of penicillin G, 100 μg/ml of gentamicin and 10 mM of pyruvate and suspended into wells of a 96-well plate (Greiner, Alphen a/d Rijn, The Netherlands). The final volume per well was 200 μl with a density of 2 ×10^6^ cells/ml. PBMCs were incubated in the absence or presence of 5 μg/ml of the lymphocyte agonist phytohemagglutin (PHA) of *Phaseolus vulgaris* (PHA-L, Roche Diagnostics GMBH, Mannheim, Germany) for 24 or 72 hours at 37°C in 5% CO_2_ atmosphere.

At the end of the incubation, the plates were centrifuged. Supernatants were kept stored at -70°C until assayed. Concentrations of IgM were measured at the end of the 72-hour incubation period; those of tumor necrosis factor alpha (TNFα) were measured at the end of the 24-hour incubation period. Measurements were done in duplicate and IgM was measured as described above. TNFα was measured by an enzyme-linked immunosorbent assay (R&D Minneapolis, MI, USA). The lower detection limit was 40 pg/ml.

### Study endpoints

The primary endpoint was the over-time changes of IgM serum levels of patients upon progression to septic shock in relation with the final outcome that is survival or 28-day mortality. The secondary study endpoint was the impact of sepsis on production of IgM from circulating lymphocytes.

### Statistical analysis

Demographic characteristics of enrolled patients were provided as percentages for qualitative variables and as means ± standard error of the mean (SE) or medians and interquartile ranges for quantitative variables. Comparisons between groups were done by the *X*^*2*^ test for qualitative variables and by ANOVA with *post hoc* Bonferroni corrections for quantitative variables.

Serum concentrations of IgM were expressed as medians and 95% confidence intervals (CI). Comparisons between groups were done by the Mann-Whitney *U* test with corrections by Bonferroni for multiple testing. Paired comparisons of serum IgM at baseline and upon progression of the same patients to a more severe stage were done by the Wilcoxon’s rank sum test. For every patient with septic shock, the area under the curve (AUC) of IgM over time for seven days was measured by the linear trapezoidal rule. Comparison between survivors and non-survivors was done by Student’s *t* test.

Concentrations of IgM and of TNFα in supernatants of PBMCs were expressed as means ± SE. Comparisons between groups were done by the Kruskal-Wallis test with corrections by Bonferroni for multiple testing. Patients were also divided into 'non-IgM’ and 'IgM-producers’ if the concentrations of IgM in supernatants of PBMCs were below the limit of detection or not. Comparisons were done by the *X*^*2*^ test.

Values of *P* below 0.05 were considered significant.

## Results

**Figure 1:**
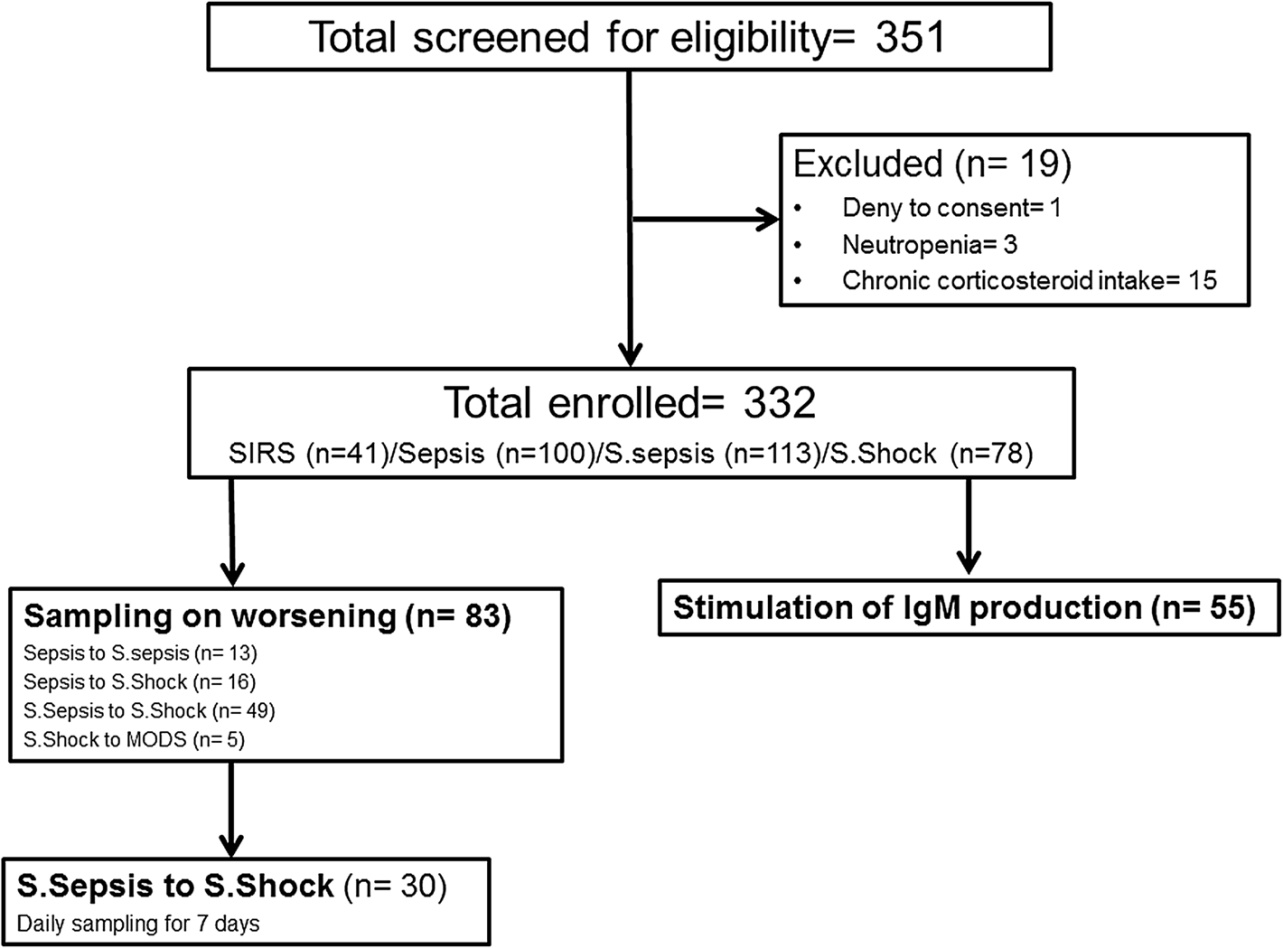
**Study flow chart.** Ig, immunoglobulin; MODS, multiple organ dysfunction syndrome; SIRS, systemic inflammatory response syndrome.

A total of 351 patients were screened and 332 were enrolled (Figure 1). The demographic characteristics of enrolled patients in relation with the severity of critical illness are shown in Table 1. As expected, acute physiology and chronic health evaluation II (APACHE II) score, WBC counts, C-reactive protein and mortality were greater in the more severe stages of sepsis.

**Table 1:** Demographic characteristics of patients enrolled in the study

	**SIRS (n= 41)**	**Sepsis (n= 100)**	**Severe sepsis (n= 113)**	**Septic shock (n= 78)**	***P***
Male/female	25/16	50/50	57/56	37/41	0.598*
Age (years, mean ± SD)	66.1 ± 10.9	66.6 ± 19.8	73.0 ± 14.1	70.0 ± 16.5	
APACHE II (mean ± SD)	5.9 ± 3.3	13.3 ± 5.9^a^	19.7 ± 6.8^b, c^	23.2 ± 6.8^d, e, f^	
WBCs (/mm^3^, mean ± SD)	11270.4 ± 4364.8	14406.8 ± 6405.6	14982.3 ± 9682.4	16442.9 ± 9446.3^d^	
Lymphocytes (/mm^3^, mean ± SD)	2298.3 ± 422.1	1942.0 ± 224.7	478.0 ± 236.2	997.3 ± 750.8	
CD19-cells (/mm^3^, mean ± SD)	182.9 ± 40.7	86.7 ± 21.5	40.9 ± 10.0	43.5 ± 19.1	
C-reactive protein (mg/l), median (IQR)	4.3 (4.9)	100.8 (140.8)^a^	141.7 (153.3)^b^	153.0 (137.5)^d^	
Infection (n %)					<0.0001*
UTI		44 (44.0)	24 (21.2)	13 (16.7)	
CAP		15 (15.0)	32 (28.3)	30 (38.5)	
IAI		24 (24.0)	17 (15.0)	8 (10.3)	
BSI		14 (14.0)	15 (13.3)	17 (21.8)	
VAP		3 (3.0)	25 (22.1)	10 (12.8)	
Acute pancreatitis	41 (100)				
Type of IAI (n, %)					0.238*
Peritonitis after gut rupture		1 (1.0)	2 (1.8)	2 (2.6)	
Acute cholecystitis		4 (4.0)	2 (1.8)	0 (0)	
Acute cholangitis		14 (14.0)	7 (6.2)	3 (3.8)	
Acute diverticulitis		2 (2.0)	0 (0)	0 (0)	
Intrabdominal abscess		3 (2.0)	5 (4.4)	3 (3.8)	
Isolated microorganisms (n, %)					<0.0001*
*Escherichia coli*	0 (0)	23 (23.0)	14 (12.4)	12 (15.4)	
*Klebsiella pneumoniae*	0 (0)	11 (11.0)	17 (15.0)	7 (8.9)	
*Acinetobacter baumannii*	0 (0)	4 (4.0)	10 (8.8)	5 (6.4)	
*Pseudomonas aeruginosa*	0 (0)	4 (4.0)	5 (4.4)	5 (6.4)	
*Proteus mirabilis*	0 (0)	7 (7.0)	4 (3.5)	1 (1.3)	
*Streptococcus pneumoniae*	0 (0)	7 (7.0)	0 (0)	3 (3.8)	
*Staphylococcus aureus*	0 (0)	0 (0)	1 (0.9)	3 (3.8)	
Other Gram-negatives	0 (0)	1 (1.0)	6 (5.3)	4 (5.1)	
Co-morbidities (n, %)					0.109*
Diabetes mellitus type 2	14 (34.2)	23 (23.0)	32 (28.3)	22 (28.2)	
Chronic obstructive pulmonary disorder	7 (17.1)	9 (9.0)	25 (22.1)	14 (17.9)	
Chronic heart failure	9 (21.9)	16 (16.0)	26 (23.0)	27 (34.6)	
Chronic renal disease	5 (12.2)	10 (10.0)	14 (12.4)	9 (11.5)	
28-day mortality (n, %)	0 (0)	14 (14.0)	52 (46.0)	46 (58.9)	<0.0001*
Hospital mortality (n, %)	0 (0)	14 (14.0)	55 (48.7)	50 (64.1)	<0.0001*

Statistically significant differences by ANOVA after *post hoc* Bonferroni corrections: ^a^sepsis vs. SIRS; ^b^septic shock vs. severe sepsis; ^c^severe sepsis vs. sepsis; ^d^septic shock vs. SIRS; ^e^severe sepsis vs. SIRS; ^f^septic shock vs. sepsis. *refers to the comparison of the distributions of the respective qualitative characteristic between SIRS, sepsis, severe sepsis and septic shock by the *X*^*2*^ test. APACHE II, acute physiology and chronic health evaluation II; *BSI,* primary bacteremia; *CAP*, community-acquired pneumonia; *IQR*, interquartile range; *IAI*, intra-abdominal infection; SD, standard deviation; SIRS, systemic inflammatory response syndrome; WBC, white blood cell; *VAP*, ventilator-associated pneumonia; *UTI*, urinary tract infection.

The study end point was the kinetics of serum IgM upon progression from severe sepsis to septic shock in relation with final outcome. To reach this end point, a three-step approach was followed (Figure 1) (a) circulating IgM was compared between critically ill patients with varying severity; (b) circulating IgM was measured at baseline and on the first day of worsening; and (c) distribution of IgM was compared over time between survivors and non-survivors from septic shock.

**Figure 2:**
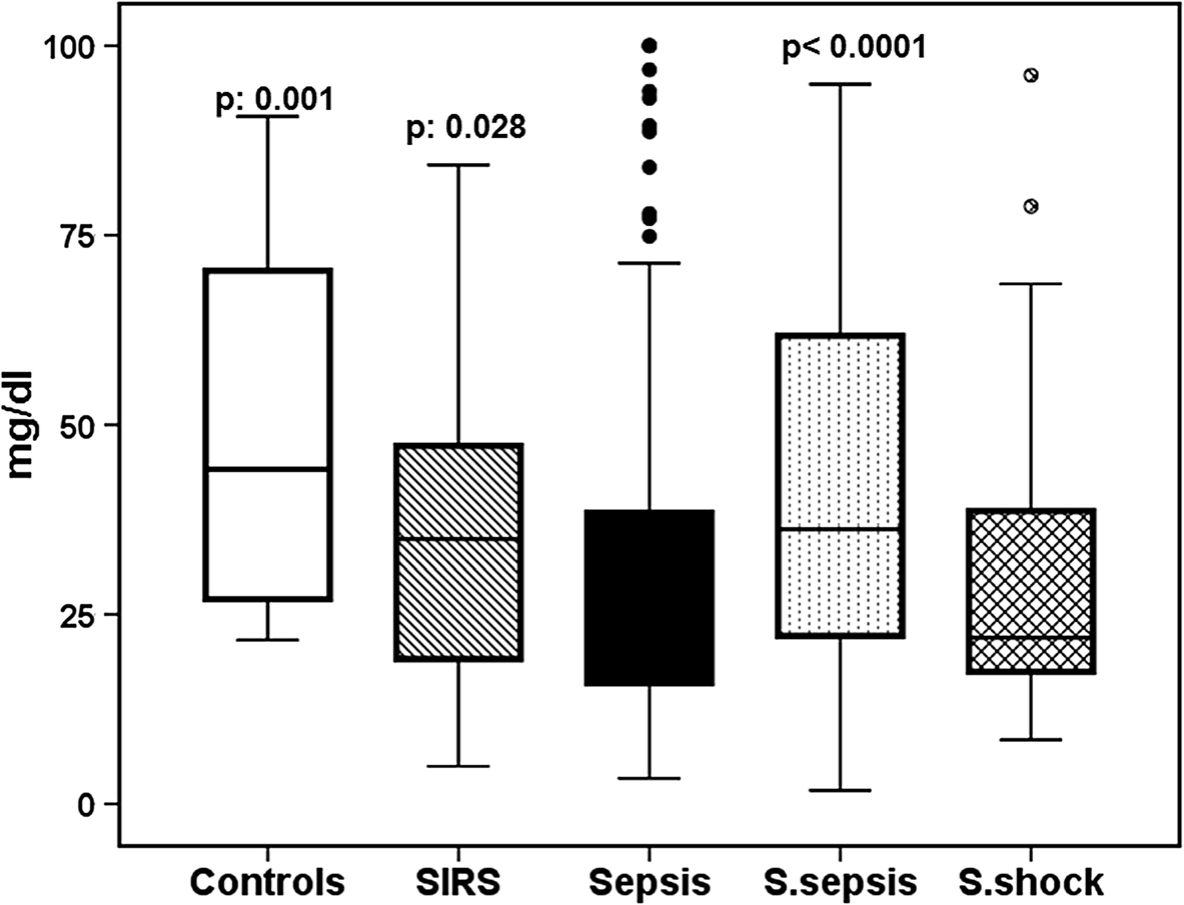
**Circulating immunoglobulin M (IgM) at various stages of severity.** IgM levels were measured in serum within the first 24 hours from diagnosis of systemic inflammatory response syndrome (SIRS) (n= 41), of sepsis (n= 100), of severe sepsis (n= 113) and of septic shock (n= 78). Serum IgM levels for healthy volunteers (n= 35) are also provided. Circles denote outliers. *P* values of comparisons with patients with septic shock after Mann-Whitney *U* test and correction for multiple comparisons are provided.

Median IgM was 44.1 mg/dl in healthy volunteers; 34.9 mg/dl in SIRS; 23.0 mg/dl in sepsis; 36.2 mg/dl in severe sepsis; and 21.9 mg/dl in septic shock. Statistical analysis showed that serum IgM was decreased in septic shock compared to healthy volunteers (*P* = 0.001), to patients with SIRS (*P* = 0.028) and to patients with severe sepsis (*P* <0.0001) but not to patients with uncomplicated sepsis (*P* = 0.754) (Figure 2).

**Figure 3:**
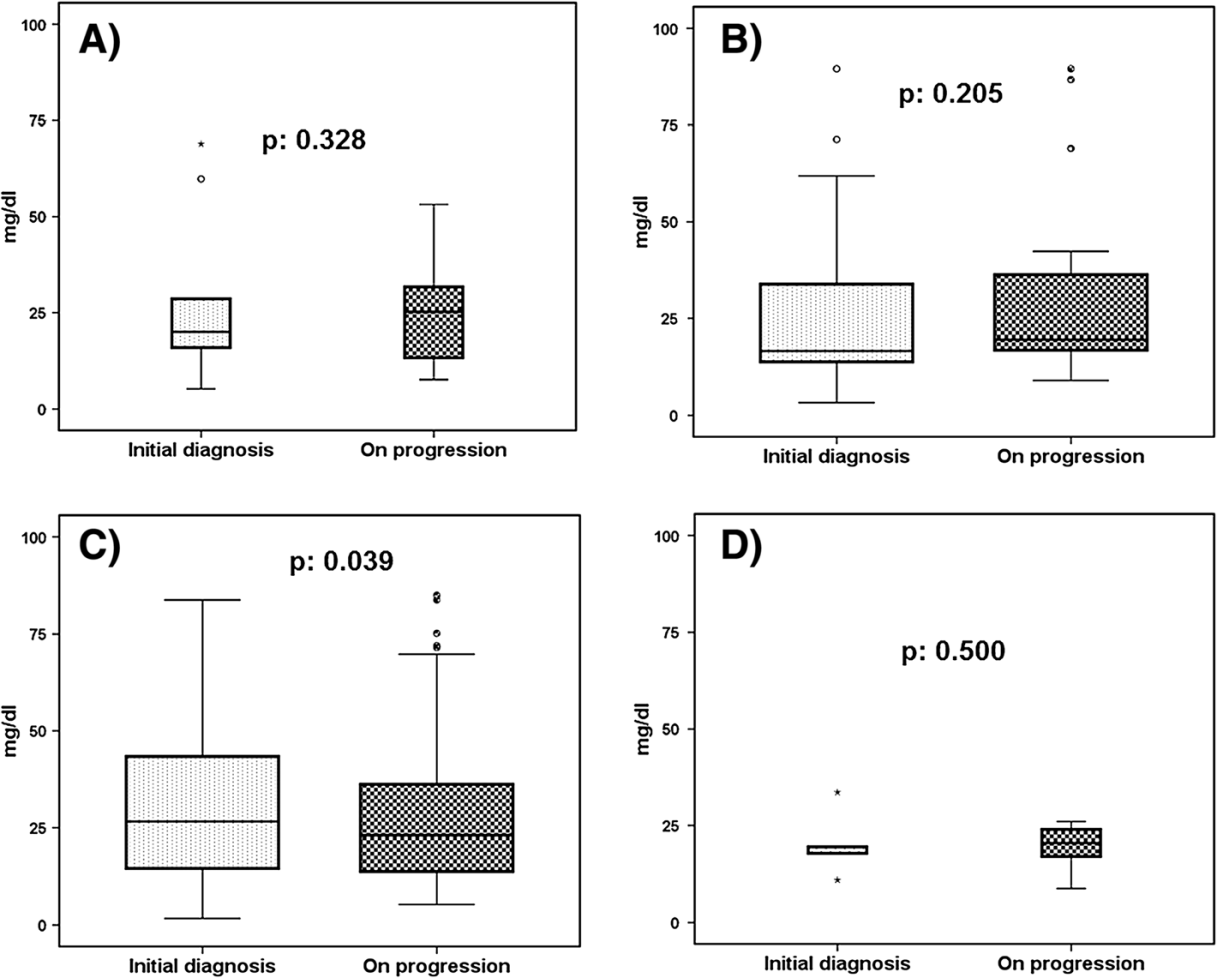
**Changes of circulating immunoglobulin M (IgM) upon worsening of sepsis.** IgM was measured: in panel **(A)** in 13 patients within the first 24 hours from diagnosis of sepsis and repeated within the first 24 hours of worsening into severe sepsis; in panel **(B)** in 16 patients within the first 24 hours from diagnosis of sepsis and repeated within the first 24 hours of worsening into septic shock; in panel **(C)** in 49 patients within the first 24 hours from diagnosis of severe sepsis and repeated within the first 24 hours of worsening into septic shock; and in panel **(D)** in 5 patients within the first 24 hours from diagnosis of septic shock and repeated within the first 24 hours of worsening into multiple organ dysfunction. *P* values of paired comparisons by the Wilcoxon rank sum test are shown. NS, non-significant.

Paired comparisons of IgM were done for 83 patients at two specific time points: on initial diagnosis and on worsening. These measurements involved: 13 patients initially diagnosed with uncomplicated sepsis who worsened into severe sepsis; 16 patients initially diagnosed with uncomplicated sepsis who worsened into septic shock; 49 patients initially diagnosed with severe sepsis who worsened into septic shock; and five patients initially diagnosed with septic shock who worsened into MODS (Figure 3). From all these paired comparisons, significant changes of circulating IgM were found only between severe sepsis and septic shock; IgM was significantly decreased upon worsening from severe sepsis into septic shock (*P* = 0.039).

**Figure 4:**
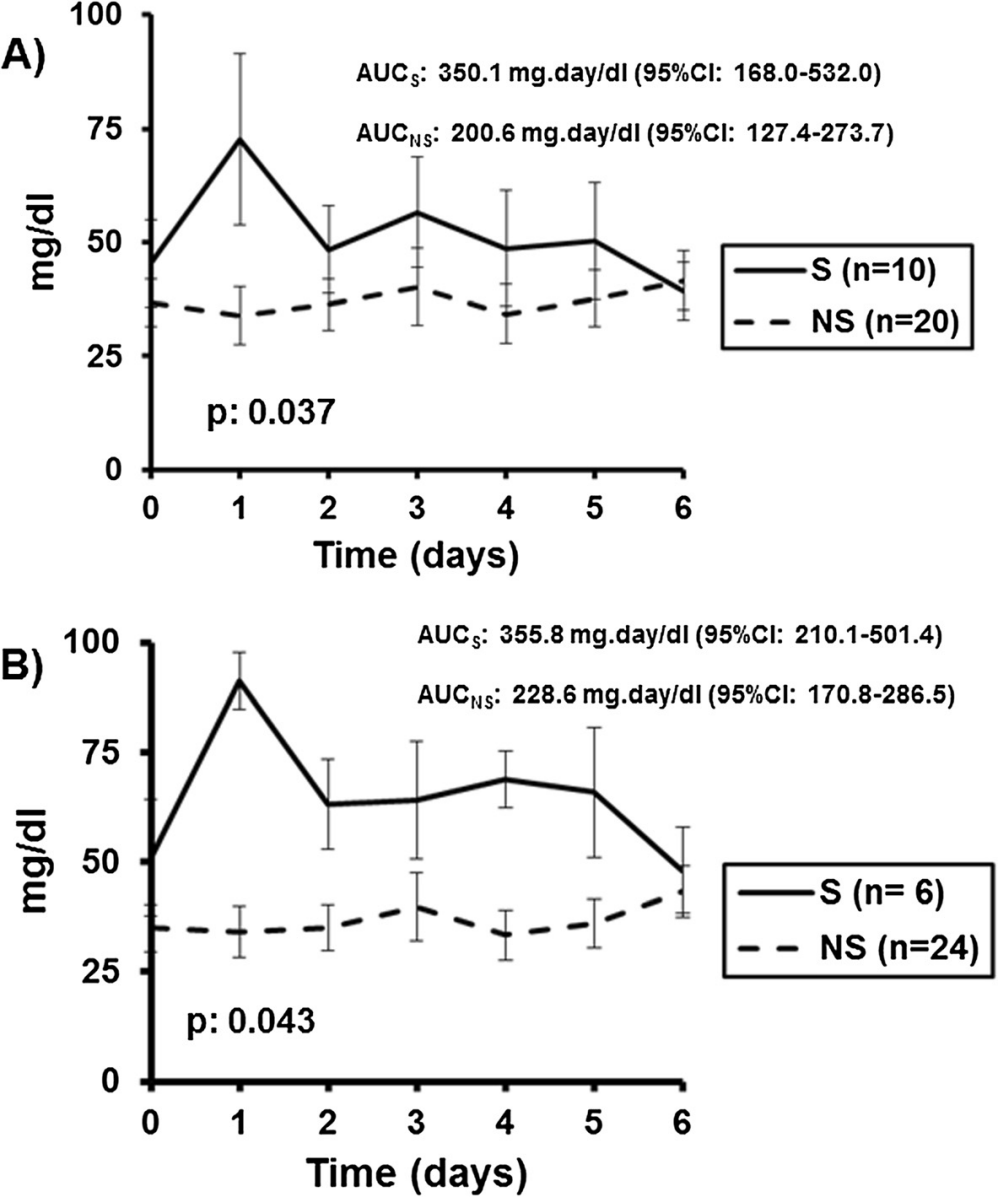
**Kinetics of immunoglobulin M (IgM) upon progression to shock.** Thirty patients with severe sepsis progressed into septic shock. Serum IgM was measured immediately after start of vasopressors (day 0) until day 6. Results are presented separately for survivors (S) and for non-survivors (NS). The area under the curve of IgM of survivors (AUC_S_) and of non-survivors (AUC_NS_) is provided. In panel **(A)** S and NS are distinguished based on their outcome after 28 days; in panel **(B)** S and NS are distinguished based on their outcome at hospital discharge. The *P* values of comparisons between AUC_S_ and AUC_NS_ by Student’s *t* test are also given. CI, confidence interval.

Within the enrolled population with septic shock, serum IgM did not differ between survivors and non-survivors (median IgM of survivors 23.1 mg/dl and of non-survivors 20.7 mg/dl, *P* = 0.442). The time curves of IgM were designed for 30 patients with severe sepsis who progressed into septic shock. Sampling was started on the day of the start of vasopressors and lasted for seven consecutive days. Separate curves were designed for survivors and non-survivors. These curves suggested that circulating IgM remained stable and at low levels in non-survivors whereas IgM of survivors increased to an early peak and then gradually decreased. As a consequence, the distribution of IgM expressed by the AUC of serum IgM over time was significantly greater for survivors than for non-survivors. This finding was similar both when outcome was assessed after 28 days and at hospital discharge (Figure 4A and 4B).

Production of IgM by PBMCs of 55 patients was also studied. From these patients, 24 had uncomplicated sepsis, 20 severe sepsis and 21 septic shock. Respective mean ± SD age was 66.5 ± 18.7, 76.4 ± 9.2 and 60.0 ± 21.4 years; mean ± SD APACHE II score was 10.7 ± 5.4, 17.4 ± 4.3 and 23.6 ± 4.8; and mean ± SD white blood cell count was 12,226.4 ± 5,262.0, 16,384.0 ± 11,294.0 and 17,130.9 ± 9,793.7/mm^3^.

**Figure 5:**
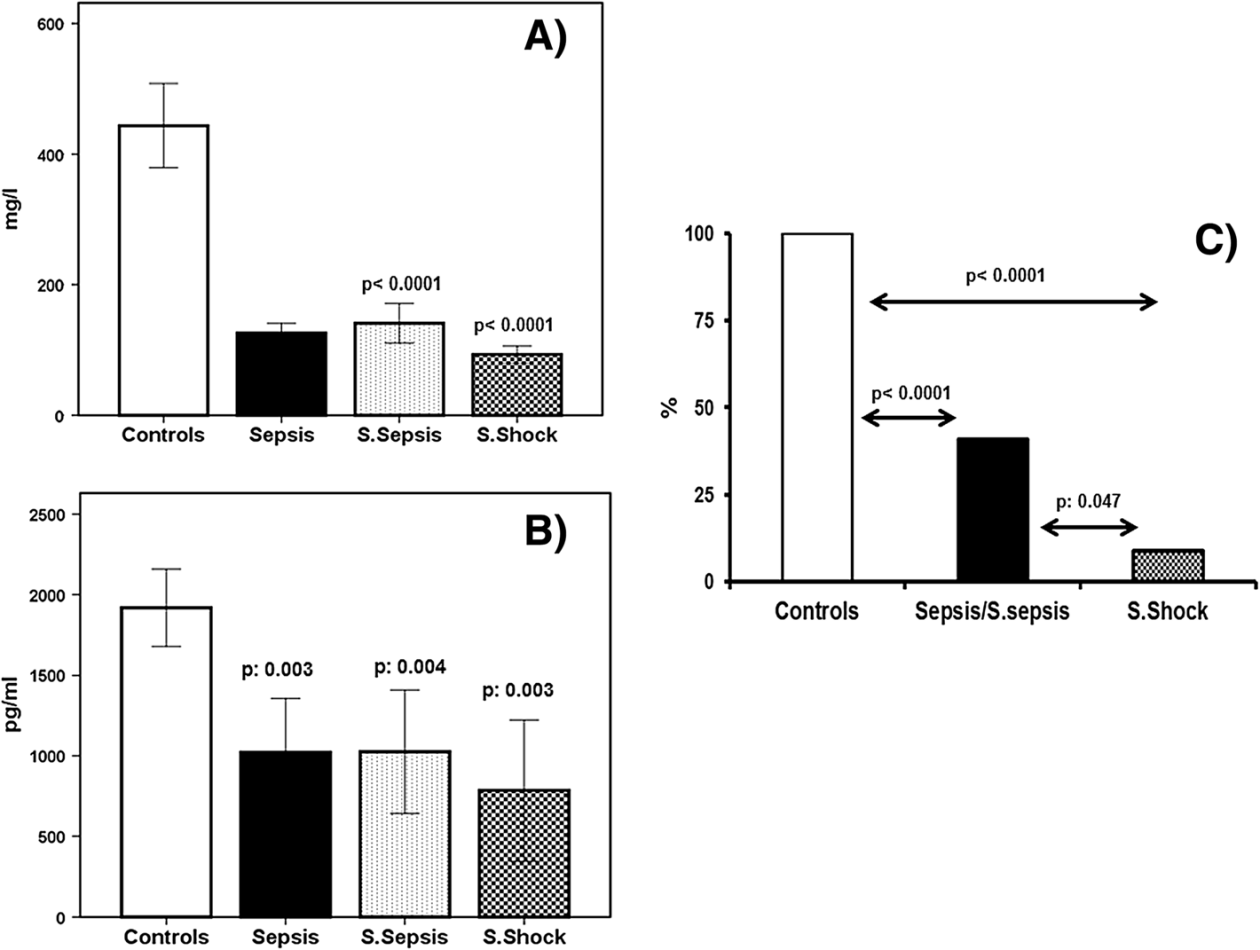
**Production of immunoglobulin M (IgM) by mononuclear cells.** Peripheral blood mononuclear cells (PBMCs) were isolated from 21 healthy volunteers, 24 patients with sepsis, 20 patients with severe sepsis and 11 patients with septic shock. PBMCs were stimulated with phytohemmaglutin (PHA) that is a selective lymphocyte agonist. Concentrations of IgM, panel **(A)** and of tumor necrosis factor alpha (TNFα), panel **(B)** were measured in supernatants. *P* values refer to comparisons with the respective production from PBMCs of healthy volunteers by the Kruskal-Wallis test after correction by Bonferroni. Production of IgM and of TNFα was below the limit of detection in supernatants of unstimulated PBMCs. Panel **(C)** shows the percentage of patients with IgM in supernatants above the limit of detection. *P* values of the indicated comparisons by the *X*^*2*^ test are provided.

High production of both IgM and TNFα was found by the PBMCs of healthy volunteers after stimulation with the selective lymphocyte agonist PHA. Production of IgM and of TNFα was significantly lower at all stages of sepsis compared with healthy controls (Figure 5A and 5B). Furthermore, the rate of 'IgM producers’ was significantly lower among patients with septic shock than among patients at all other sepsis stages (Figure 5C).

## Discussion

The present study is the largest cohort to the best of our knowledge that describes the kinetics of circulating IgM in sepsis. Analysis indicates that the decrease of IgM is a predominant characteristic when a patient with severe sepsis develops septic shock. Close monitoring from the start of vasopressors shows that the distribution of IgM is greater in survivors than in non-survivors from septic shock.

These conclusions are based on the multilevel approach of the current study; at first, comparisons between SIRS, sepsis, severe sepsis and septic shock indicated that septic shock is the stage of critical illness with the lower circulating IgM; then measurements at distinct time points that is, upon initial diagnosis and upon worsening showed that circulating IgM decreases specifically upon progression from severe sepsis to septic shock; and finally, intense monitoring of IgM after the start of vasopressors revealed a relationship between lacking distribution of IgM and unfavorable prognosis.

IgM levels in patients with septic shock are reported in two more studies. In the first study [14], IgM was decreased in 21 patients with septic shock. Patients were followed up every 48 hours for five days and they were divided into those with hypo IgM concentrations and normal IgM concentrations. No differences were found between survivors and non-survivors. In the second study [15], low IgM levels were reported in the plasma of 62 patients with septic shock. The IgM concentrations reported by the authors of this study were within the range of concentrations reported in our study. However, the authors failed to define any differences in circulating IgM between survivors and non-survivors [15]. In their study, blood samplings of days 1 and 2, of days 3 and 4 and of days 5 to 7 were reported together which did not allow measurement of the distribution of circulating IgM as this was done in our study.

IgM is a polyvalent immunoglobulin circulating as a pentamer [3]. It opsonizes bacteria and primes phagocytosis by neutrophils; it binds and inactivates endotoxins of Gram-negative bacteria and exotoxins of Gram-positive cocci; and it also binds and inactivates proinflammatory host mediators like cytokines. Its role is underscored by models of experimental sepsis in mice; survival is prolonged after induction of sepsis through cecal ligation and puncture (CLP) within the animals that possess the highest potential for IgM-primed phagocytosis [16]. Recent data coming both from rodents and humans suggest that release of IgM is primed by a new subset of B lymphocytes known as IRA (innate response activator) B cells. These cells belong to the innate defense system, they contain large cytoplasmic stores of IgM antibodies and they are the main effectors of the rapid release of IgM. IRA B cells are depleted in experimental sepsis and this leads to early death [17]. The evidence coming from experimental animal data may help explain the importance of the *ex vivo* production of IgM from our patient population. All patients produced much lower IgM than healthy volunteers; this defect was exaggerated in septic shock. Our findings lead to the hypothesis that during severe sepsis lymphocytes are hypofunctional for IgM production but high circulating IgM compensates for the patient’s needs; once septic shock develops circulating IgM is fully consumed and lymphocytes are completely anergic for any IgM production.

Two major limitations of the current study should, however, be acknowledged: (a) the lack of explanation why septic shock is a specific condition where circulating IgM is depleted. It is most probable that this is related with the consumption of circulating IgM during sepsis worsening and with the inability of B lymphocytes for IgM production; and (b) the lack of explanation from our findings why circulating IgM does not differ between uncomplicated sepsis and septic shock.

## Conclusions

The present study managed to identify specific changes of the kinetics of circulating IgM that are related with final outcome. These occur when patients with severe sepsis progress to septic shock. In these patients, the distribution of IgM is lower among non-survivors. These findings may guide the design of future RCTs for the management of septic shock.

## Key messages

● Serum levels of IgM are significantly decreased in septic shock but not in severe sepsis.

● Dramatic changes of serum IgM occur when patients at severe sepsis progress into septic shock. In these cases, the distribution of IgM is lower among non-survivors.

● Circulating lymphocytes of patients render anergic for the production of IgM.

## Abbreviations

APACHE II: Acute physiology and chronic health evaluation II; ARDS: Acute respiratory distress syndrome; BSI: Primary bacteremia; CAP: Community-acquired pneumonia; IAI: Intra-abdominal infection; IgM: Immunoglobulin M; IRA: Innate response activator; MODS: Multiple organ dysfunction syndrome; PBMC: Peripheral blood mononuclear cell; PHA: Phytohemagglutin; RCT: Randomized controlled trial; SIRS: Systemic inflammatory response syndrome; TNFα: Tumor necrosis factor alpha; UTI: Acute pyelonephritis; VAP: Ventilator-associated pneumonia; WBC: White blood cell.

## Competing interest

The authors declare that they have no competing interests related to this submission.

## Authors’ contributions

EJGB designed the study and performed the analysis, wrote the manuscript and agreed to the final submitted version. EA, ML, IP, NKG, IT, MB, KS and AK provided clinical data, drafted the manuscript and agreed to the final submitted version. MG and TK conducted laboratory experiments, drafted the manuscript and agreed to the final submitted version. MAK validated the statistical analysis, drafted the manuscript and agreed to the final submitted version. AA participated in study design and interpretation of data, drafted the manuscript and agreed to the final submitted version. All authors read and approved the final manuscript.
